# Youth Smoking: Toward a Better Understanding and Realistic Solutions

**DOI:** 10.1186/1617-9625-2-3-117

**Published:** 2004-09-15

**Authors:** Daniel R Longo

**Affiliations:** 1 Editor-in-Chief, Columbia, Missouri, USA

## The problem

Clearly all readers of this journal understand the tremendous public health problem of youth smoking. Regardless, there are several facts worth repeating. We know, as Steve Sussman and colleagues in this issue of the journal remind us citing Flay (1993): "Most regular adolescent tobacco users are likely to continue into adulthood." If one were to view today's American Cancer Society website we would also be reminded of the most recent findings from the National Youth Tobacco Survey regarding smoking among American Youth. They write:

• Nationwide, about 28% of high school students reported using some type of tobacco (cigarette, cigar, pipe, bidi, kretek, or smokeless tobacco) on at least 1 of the 30 days before the survey.

• Over one-fifth (23%) of high school students smoked cigarettes on at least 1 of the 30 days before the survey in 2002, down from the recent high of 36% in 1997.

• About 6% of high school students reported using smokeless tobacco at least once in the 30 days before the survey. Regardless of race, male students were much more likely to use smokeless tobacco than female students.

• About 12% of high school students had smoked cigars in the preceding 30 days. Male students (17%) were more likely to smoke cigars than female students (6%).

(Reported from American Cancer Society web site: )

Obviously this problem is worldwide, and as such, this epidemic is one of the major concerns of the International Society for the Prevention of Tobacco Induced Diseases. The U.S. statistics reported above are similar to those in many developed countries, while those in the developing world are even more staggering. Worldwide statistics from the World Health Organization indicate and report an "upward trend in tobacco initiation and use among children as tobacco is available to children in many countries, even countries with legal prohibitions against tobacco sales to those younger than the age of accountability."

Let us consider data from the United Kingdom and the Statistical Bulletin that reports for 2003:

• In 2001, 27% of adults aged 16 and over smoked cigarettes in England; 28% of men and 25% of women.

• The prevalence of cigarette smoking among adults has dropped substantially since 1980 (from 39%), although it levelled off in the 1990s.

• In 2001, the prevalence of cigarette smoking continued to be higher for people in manual than non-manual socio-economic groups (32% compared with 21%).

• In 2001, 66% of smokers in England wanted to give up smoking.

• In 2002, 10% of children aged 11–15 smoked cigarettes regularly; 9% of boys and 11% of girls.

• More than 120,000 deaths were caused by smoking in the UK in 1995; that is, one in five of all deaths.

(Reported from: Statistical Bulletin 2003/21: Statistics on smoking: England, 2003 Toward Possible Solutions.)

## Toward realistic solutions

There will never be one "magic bullet" to solve this problem. Thus, we must evaluate various approaches that may contribute to the solution and may work for specific populations. Sussman and colleagues' paper presents the "Project EX research program" that involves novel activities (e.g., "talk show enactments," games, and alternative medicine-type activities such as yoga and meditation) in combination with motivation enhancement and cognitive-behavioral strategies to motivate and instruct in cessation initiation and maintenance efforts. They report outcomes of the first experimental trial of Project EX, a school-based clinic program. A second EX study, a multiple baseline single group pilot study design in Wuhan, China is described next. A second experimental trial, which tested EX with nicotine gum versus a natural herb, is also described, as well as a third experimental trial that tested a classroom prevention/cessation version of EX. The results are very promising – the intent-to-treat quit rate for Project EX is approximately 15% across studies, double that of a standard care comparison, and effects last up to six months post-program at regular and alternative high schools. These authors suggest that future cessation work might expand on this work. We present this challenge to our readers.

Nyi Nyi Naing and colleagues report a cross-sectional study that was conducted to identify the factors related to smoking habits of adolescents among secondary school boys in Kelantan state, Malaysia. Findings indicate that peer influence was the major reason students gave for taking up the habit, while religion was most often indicated by non-smokers as their reason for not smoking. These authors find that the use of mass media was indicated as the best information source for the students to acquire knowledge about negative aspects of the smoking habit. The authors recommend that anti-smoking campaigns with an emphasis on the religious aspect should start as early as in primary school. Again, we challenge our research community to replicate this study in other countries.

While numerous interventions aimed at decreasing and preventing youth smoking have been proposed and evaluated by researchers internationally, far more work needs to be done. It is anticipated that the studies in this issue of the journal will contribute to those goals. In each issue of the journal we will highlight one or more articles with an editorial to help place those articles and their findings in the context of issues of current concern. In addition, several other excellent articles are included in this issue.

## Competing interests

The authors declare that they have no competing interests.

